# Therapeutic Prospects of *Undaria pinnatifida* Polysaccharides: Extraction, Purification, and Functional Activity

**DOI:** 10.3390/md23040163

**Published:** 2025-04-08

**Authors:** Kit-Leong Cheong, Wenjie Chen, Min Wang, Saiyi Zhong, Suresh Veeraperumal

**Affiliations:** 1 Guangdong Provincial Key Laboratory of Aquatic Product Processing and Safety, College of Food Science and Technology, Guangdong Ocean University, Zhanjiang 524088, China; klcheong@gdou.edu.cn (K.-L.C.); chenwj0610@163.com (W.C.); 2 College of Coastal Agriculture Sciences, Guangdong Ocean University, Zhanjiang 524088, China; wangmin@gdou.edu.cn; 3 Department of Biology, College of Science, Shantou University, Shantou 515063, China

**Keywords:** *Undaria pinnatifida*, polysaccharides, isolation, purification, biological activity

## Abstract

*Undaria pinnatifida*, an edible brown seaweed that is widely consumed in East Asia, has gained increasing recognition for its health benefits. Among its bioactive compounds, polysaccharides have attracted significant attention due to their diverse biological activity. This review provides a comprehensive overview of recent advancements in the extraction, purification, structural characterization, and bioactivity of *U. pinnatifida* polysaccharides. We discuss state-of-the-art extraction techniques, including ultrasound-assisted, microwave-assisted, and enzyme-assisted extraction, as well as purification strategies such as membrane separation and chromatographic methods. Furthermore, we highlight their potential biological activity, including antioxidant, immunomodulatory, anticancer, gut health-promoting, and anti-hyperglycemic effects, along with their underlying mechanisms of action. By summarizing the latest research, this review aims to provide valuable insights into the development and application of *U. pinnatifida* polysaccharides in functional foods and pharmaceuticals.

## 1. Introduction

*Undaria pinnatifida*, an edible brown seaweed that is widely consumed in East Asia, is valued for both its traditional dietary role and extensive health benefits [1]. The growing global interest in marine-based nutraceuticals and functional foods has positioned *U. pinnatifida* as a vital bioresource in the food, pharmaceutical, and cosmetic industries [2,3]. Its widespread cultivation in marine aquaculture is attributed to its rapid growth, high biomass yield, and sustainability. As research on marine bioresources advances, *U. pinnatifida* stands out in marine biotechnology due to its renewable nature, diverse biochemical composition, and broad industrial applications. It is rich in bioactive compounds, including polysaccharides, proteins, vitamins, minerals, and polyphenols, which contribute to its functional properties [4,5,6]. Among these, polysaccharides have garnered particular interest due to their diverse and potent biological activity.

Polysaccharides are vital bioactive compounds with diverse physiological roles and substantial potential across the food, pharmaceutical, and biomedical industries [7,8]. As naturally occurring macromolecules, they possess a broad spectrum of biological activity, including antioxidant, anti-inflammatory, immunomodulatory, and antimicrobial properties [9,10,11]. Their functional characteristics are largely determined by their structural complexity, which arises from variations in the monosaccharide composition, glycosidic linkages, and branching patterns [12,13]. The *U. pinnatifida* polysaccharides (UPPs) exhibit distinct structural and compositional differences compared to those in terrestrial plants. UPPs primarily comprise fucoidan, alginate, and laminarin—three unique polysaccharides that are rarely present in land plants [14,15]. These compounds have been extensively studied for their roles in immune regulation, gut microbiota modulation, and metabolic health support. Polysaccharides are generally biocompatible, biodegradable, and exhibit low toxicity, making them highly desirable as natural health-enhancing agents [16,17,18]. Advances in extraction and purification techniques have significantly improved the isolation of bioactive polysaccharides, promoting their application in functional foods, nutraceuticals, and innovative drug formulations [19,20]. As research continues to elucidate their mechanisms of action, polysaccharides are being increasingly recognized as essential components in next-generation health products, reinforcing their significance as multifunctional bioactive compounds.

This review aims to compile existing knowledge and emphasize recent progress in the extraction, purification, and biological functions of UPPs. It specifically analyzes different isolation and purification methods employed to obtain these bioactive molecules. Furthermore, the review investigates the structural characteristics of key UPPs—fucoidan, laminarin, and alginate—and their relationship with biological activity. A key objective is to summarize the wide-ranging biological effects of these polysaccharides, including their antioxidant, anti-inflammatory, immunomodulatory, gut-microbiota-modulating, and metabolic-health-supporting properties. By integrating current research and offering insights into future directions, this review serves as a valuable reference for scientists and industry experts interested in the potential of UPPs as bioactive compounds with promising health benefits and commercial applications.

## 2. Physicochemical and Structural Features of *U. pinnatifida* Polysaccharides

### 2.1. Fucoidan

Fucoidan derived from *U. pinnatifida* is a sulfated polysaccharide primarily composed of α-(1→3) and α-(1→4)-linked L-fucose units, with sulfate groups typically positioned at C-2, C-3, or C-4 [21,22]. The degree and pattern of sulfation play a crucial role in its bioactivity, influencing its anticoagulant, anti-inflammatory, and immunomodulatory properties [9]. Higher sulfation levels enhance fucoidan’s water solubility and biological interactions [23]. Its physicochemical properties and structural characteristics vary depending on the seaweed species, extraction methods, and environmental conditions [24]. The molecular weight of fucoidan typically ranges from 10 to 500 kDa [25], with low-molecular-weight fractions often demonstrating higher bioactivity due to improved solubility and cellular absorption [26]. Fucoidan’s monosaccharide composition primarily consists of L-fucose, with minor amounts of galactose, mannose, xylose, and uronic acids [27].

### 2.2. Alginate

Alginate is a linear anionic polysaccharide composed of β-(1→4)-linked mannuronic acid (M) and guluronic acid (G) residues, arranged in homopolymeric (M-blocks and G-blocks) and heteropolymeric (MG-blocks) sequences [28]. Its physicochemical properties are largely determined by the M/G ratio, which influences its gelation ability, viscosity, and mechanical strength. Alginate is highly soluble in neutral and alkaline conditions but forms gels in the presence of divalent cations, such as calcium (Ca^2+^), through ionic crosslinking between G-blocks [29]. The molecular weight of alginate ranges from 50 to 500 kDa, affecting its viscosity and functional properties in biomedical and food applications [30,31]. The spatial arrangement of mannuronic and guluronic acids significantly impacts the rigidity and elasticity of alginate gels—high-G alginates form strong, brittle gels, while high-M alginates produce softer, more flexible gels [32]. Additionally, the degree of acetylation modulates alginate’s solubility and rheological properties, influencing its interactions with proteins and other biopolymers [33].

### 2.3. Laminarin

Laminarin is a low-molecular-weight β-glucan, primarily composed of a β-(1→3)-linked glucan backbone with occasional β-(1→6)-linked branches [34,35]. These branching patterns influence its solubility, structural conformation, and biological activity. The molecular weight of laminarin typically ranges from 2 to 10 kDa, depending on the extraction method and seaweed growth conditions [36]. Laminarin is predominantly composed of glucose, with minor amounts of mannose, galactose, and, in some cases, sulfate or acetyl groups, which can modify its physicochemical properties and bioactivity [37]. The β-(1→3)-linked backbone facilitates interactions with immune receptors, contributing to its immunomodulatory and antioxidant effects [38]. These structural features make laminarin a promising candidate for applications in functional foods, pharmaceuticals, and cosmetics.

## 3. Extraction of *U. pinnatifida* Polysaccharides

The extraction process plays a vital role in determining the yield, purity, and structural stability of UPPs, directly affecting their bioavailability and biological activity. An optimized extraction technique not only enhances polysaccharide recovery but also maintains or improves their structural characteristics, ensuring greater bioefficacy [39]. Conversely, suboptimal conditions may cause polysaccharide degradation, reduced solubility, and altered biological properties, restricting their potential applications in pharmaceuticals, nutraceuticals, and cosmetics [40]. Traditionally, UPP extraction has primarily relied on conventional techniques such as hot water and acid/base extraction due to their simplicity and cost efficiency. Hot water extraction involves immersing dried or fresh *U. pinnatifida* in heated water for several hours to break down the seaweed’s cell walls and release polysaccharides [41]. For instance, one study extracted polysaccharides from pre-treated *U. pinnatifida* powder (20 g) using distilled water at a 1:20 ratio (g/mL) under reflux for five hours, yielding 6.64% polysaccharides based on the dry weight [42]. Although hot water extraction is environmentally friendly, it presents certain drawbacks, including lengthy processing times and relatively low extraction efficiency.

Acid extraction methods are commonly utilized to improve the recovery of polysaccharide fractions, particularly sulfated polysaccharides such as fucoidans [43]. This approach uses dilute acids to degrade cell walls, facilitating the release of polysaccharides. For example, previous research successfully extracted fucoidan using 0.83 mmol/L citric acid at 95 °C for 40 min [44], while another study employed 0.1 mol/L HCl diluted tenfold for 8 h at 45 °C [14]. However, a major drawback of acid extraction is depolymerization, which can lower the molecular weight of polysaccharides and potentially alter their bioactivity [45]. Comparative analyses suggested that acid extraction at 80 °C for 24 h resulted in the highest total sugar content (38.35%), followed by alkali extraction (37.43%) and water extraction (11.01%). Interestingly, hot water extraction produced the highest molecular weight (300 kDa), indicating the better preservation of the polysaccharide structure. In contrast, acid and alkali extractions led to significantly reduced molecular weights of 88 kDa and 110 kDa, respectively, which may impact their biological activity and functional properties [46].

Enzyme-assisted extraction (EAE) is an advanced and targeted approach that utilizes hydrolytic enzymes, such as cellulase, pectinase, and alginate lyase, to break down the structural components of *U. pinnatifida* cell walls, thereby enhancing polysaccharide release [47]. Unlike conventional techniques that depend on high temperatures or harsh chemical treatments, EAE operates under milder conditions, helping to maintain the structural integrity and bioactivity of the extracted polysaccharides [48]. In one study, *U. pinnatifida* powder was mixed with water and 0.5 mL of Celluclast enzyme and then incubated at 50 °C for 24 h in a shaking incubator. The extracted UPPs contained fucose (52.3%), galactose (44.5%), and sulfate content of 30.4% [49].

Microwave-assisted extraction (MAE) is an innovative technique that utilizes microwave radiation to rapidly heat the extraction medium, facilitating the breakdown of *U. pinnatifida* cell walls and enhancing the release of bioactive polysaccharides [50]. Unlike conventional heating methods that rely on external heat transfer, MAE generates heat through direct interaction between microwaves and polar molecules, ensuring uniform heating, shorter extraction times, and improved efficiency [51]. The rapid thermal effect disrupts hydrogen bonds and weakens polysaccharide–cell wall interactions, resulting in higher yields [52]. In a study by Zhong et al., depigmented *U. pinnatifida* powder was mixed with deionized water at a 1:30 (*m*/*v*) ratio and subjected to MAE at microwave power of 600 W and 70 °C for 20 min, yielding 3.53% crude polysaccharides [53]. Another study reported that the highest fucoidan yields were obtained at 150 °C for 30 min. However, excessive microwave temperatures caused increased fucoidan degradation; for example, at 140 °C, the fucoidan had a molecular weight of approximately 13 kDa, whereas, at 100 °C, it retained a molecular weight of 240 kDa [54].

**Figure 1:**
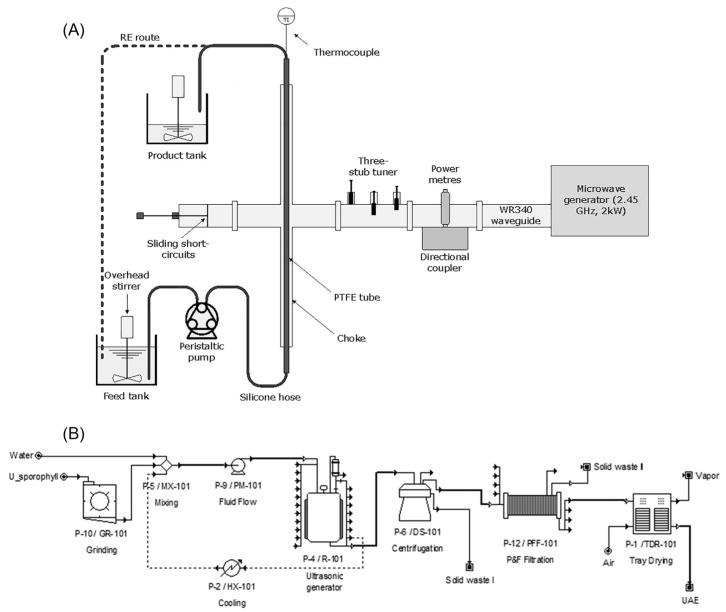
The proposed flowsheet for the pilot-scale extraction of *U. pinnatifida* using microwave-assisted extraction (**A**), adapted with permission from reference [59], copyright 2020, Elsevier; ultrasound-assisted extraction (**B**), adapted with permission from reference [60], copyright 2020, Elsevier.

Ultrasound-assisted extraction (UAE) is a highly efficient and environmentally friendly technique that utilizes acoustic cavitation to enhance polysaccharide recovery [55]. High-frequency sound waves generate microbubbles that collapse rapidly, creating localized high pressure and temperatures [56]. This physical disruption breaks down seaweed’s cell walls, increasing their permeability and facilitating the release of intracellular polysaccharides. In a study by Lee et al., *U. pinnatifida* sporophyll was extracted using UAE under optimized conditions, namely 1080 W ultrasonic power, an 80% amplitude, a 20 kHz frequency, a 30 °C extraction temperature, and an 8 h extraction time, resulting in a 31.91% UPP yield [57]. Similarly, Song et al. compared UAE with conventional thermal extraction and found that UAE with HCl (pH 2) at an 80% amplitude for 6 h achieved a 33% extraction yield. In contrast to traditional thermal extraction, UAE demonstrated higher efficiency and a significantly shorter extraction time [58]. As shown in Figure 1, the potential for the pilot-scale production of UPPs using UAE and MAE is promising due to their efficiency in enhancing yields and reducing the extraction time [59,60]. Pilot-scale UAE systems can be designed with continuous-flow reactors to improve the processing efficiency, following an industrialization flowchart divided into grinding, extraction, separation, and drying processes. Meanwhile, large-scale MAE can incorporate temperature-controlled microwave reactors to ensure uniform heating and prevent polysaccharide degradation.

## 4. Purification of *U. pinnatifida* Polysaccharides

### 4.1. Preliminary Purification

The purification of UPPs is a crucial step in enhancing their biological activity and functional properties. Crude UPP extracts often contain impurities, such as proteins, polyphenols, salts, and low-molecular-weight compounds, which can interfere with their bioactivity, stability, and safety [61]. These contaminants may reduce the efficacy of UPPs in pharmaceutical, nutraceutical, and cosmetic applications by compromising their structural integrity and physiological interactions. Purification not only improves the consistency and reproducibility of UPP-based formulations but also enhances their specific biological activity.

**Figure 2:**
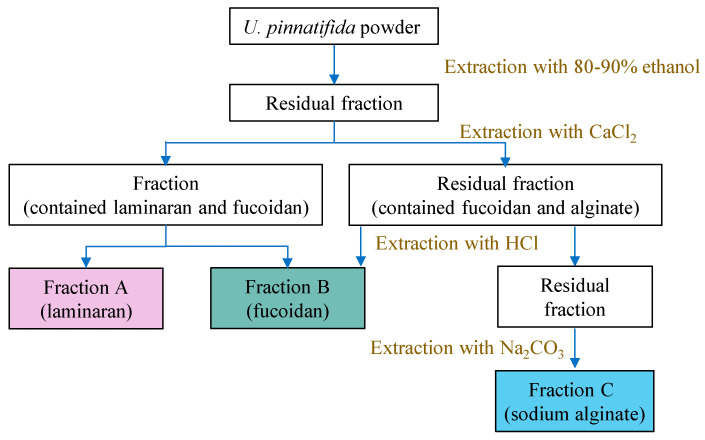
Sequential extraction of different polysaccharide types from *U*. *pinnatifida* with different solvents.

In a study, *U. pinnatifida* powder was initially extracted with 80–90% (*v*/*v*) ethanol at room temperature for 12 h or at 70 °C for 5 h to remove pigments and proteins [62]. The residual seaweed was then treated with 2% (*w*/*v*) CaCl_2_ at 70 °C for three cycles of 3 h each to separate the laminaran and fucoidan fractions. Fucoidan (fraction B) was extracted from the residual seaweed using 0.01 mol/L HCl (pH 2) at 70 °C (Figure 2) [62]. Alternatively, ultrafiltration could be used to separate fucoidan and laminaran based on their molecular weights. A 10 kDa molecular weight cutoff membrane was employed to collect Fraction B as fucoidan, while a 2 kDa molecular weight cutoff membrane separated the lower-molecular-weight laminaran (fraction A) [63]. Alginate (fraction C) was extracted using 3% (*w*/*v*) Na_2_CO_3_ at 70 °C, followed by centrifugation. The solute in the supernatant was then precipitated using ethanol to obtain sodium alginate.

Ethanol precipitation is a widely used technique that separates polysaccharides from proteins, salts, and low-molecular-weight compounds based on their differential solubility [64]. In one method, the crude polysaccharide extract was mixed with 99.5% ethanol at a 2:1 ratio and incubated at 4 °C to precipitate the polysaccharides, which were then collected by centrifugation [57]. Another approach involved adding four times the volume of anhydrous ethanol to the extraction supernatant, followed by centrifugation, redissolution, and freeze-drying to yield UPPs. While this method is simple and cost-effective, it lacks selectivity and may result in the co-precipitation of unwanted substances [65].

Membrane filtration and ultrafiltration are commonly used to remove small molecules such as salts and free sugars, using semipermeable membranes with controlled molecular weight cutoffs [66,67]. For instance, crude UPPs were passed through ultrafiltration membranes with molecular weight cutoffs of 300 kDa and 10 kDa, resulting in high-molecular-weight fucoidan (258.7 kDa) and low-molecular-weight fucoidan (ranging from 1.32 to 1330 kDa) [44]. In another study, the extraction supernatant was ultrafiltered through a 10 kDa cutoff membrane for five cycles and then concentrated, yielding UPPs with molecular weights between 202.41 kDa and 8146.33 kDa [68]. However, these techniques can be time-consuming and may lead to the loss of low-molecular-weight polysaccharides.

### 4.2. Chromatographic Purification

**Figure 3:**
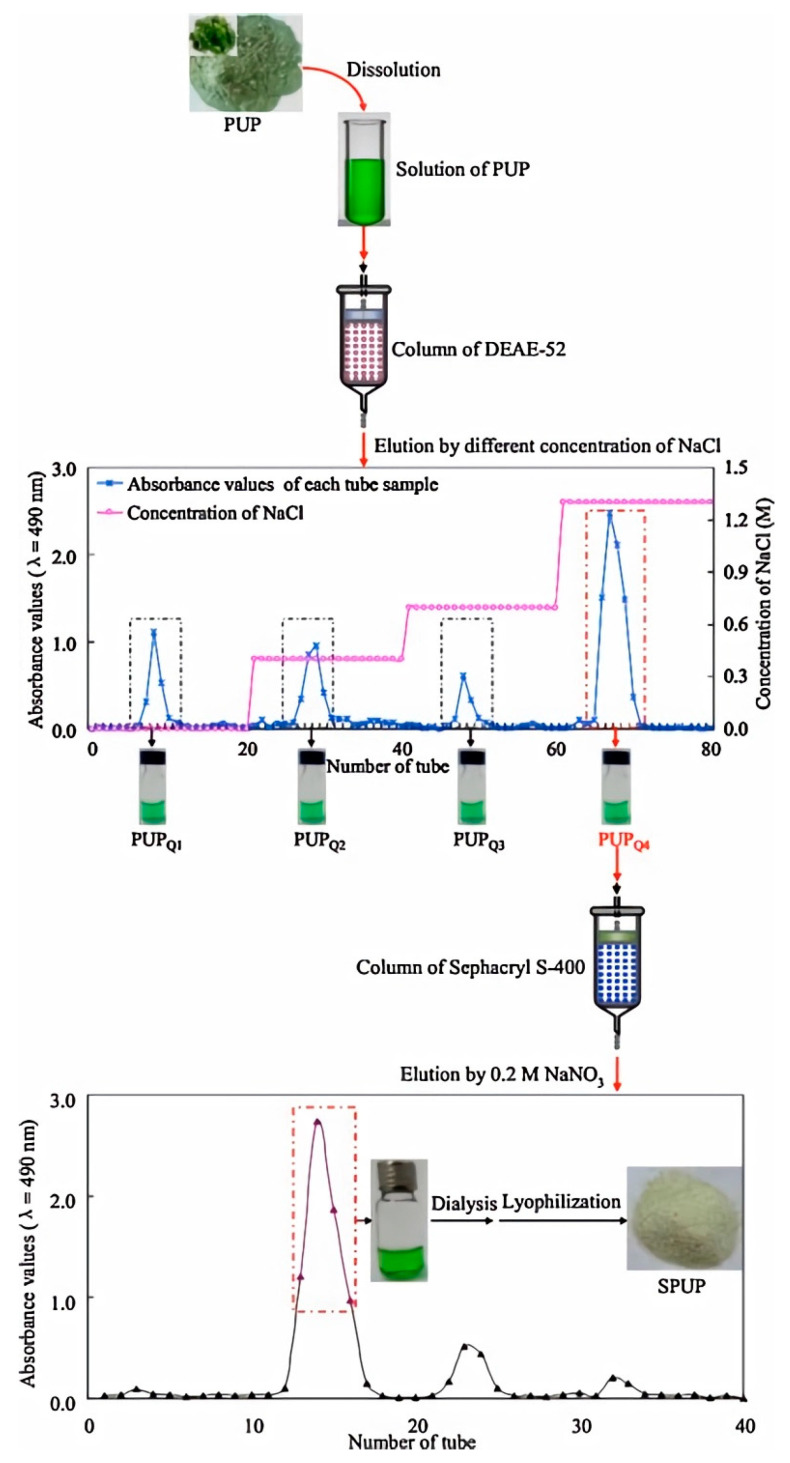
Purification flow diagram for UPPs, which are first separated onto a DEAE-52 cellulose column, subjected to sequential elution with distilled water and gradient elution with NaCl solutions, and then further purified on a Sephacryl S-400 gel filtration column. Adapted with permission from reference [42], copyright 2016, Elsevier.

Ion exchange chromatography is a technique that employs charged resins to selectively isolate UPPs based on their sulfate and carboxyl group content, thereby enhancing the purity of specific bioactive fractions [69]. However, this method requires precise control over the pH and ionic strength, making it both labor-intensive and costly [70]. In one study, crude polysaccharides were separated using a DEAE-cellulose column and eluted with NaCl solutions of varying concentrations (0–4 mol/L), yielding four distinct polysaccharide fractions: UPF1, UPF2, UPF3, and UPF4. Among these, UPF3 exhibited the highest sulfate content (39.79%) and contained the highest concentrations of fucose (143 μg/mg) and galactose (208 μg/mg) [71]. Size exclusion chromatography (SEC) is another widely employed method that separates polysaccharides based on their molecular sizes to obtain high-purity fractions [72]. However, SEC is often limited by its scalability and lower throughput [73]. In a study by Han et al., crude UPPs were initially purified using DEAE-cellulose through sequential elution with distilled water, followed by a NaCl gradient (0–2.5 mol/L). The fraction eluted with 1.3 mol/L NaCl was then further purified using Sephacryl S-400 SEC, yielding a heteropolysaccharide with a monosaccharide composition of fucose, glucose, and galactose in a molar ratio of 27.15:19.34:53.51 and a molecular weight of 97.9 kDa [42]. Both ion exchange chromatography and size exclusion chromatography are valuable techniques in achieving high-purity UPP fractions. A schematic diagram illustrating these purification methods is shown in Figure 3. While both ion exchange and size exclusion chromatography are effective purification techniques, they face limitations such as low selectivity, process inefficiencies, and potential yield losses. These challenges underscore the need for more advanced, high-throughput, and eco-friendly purification approaches.

**Figure 4:**
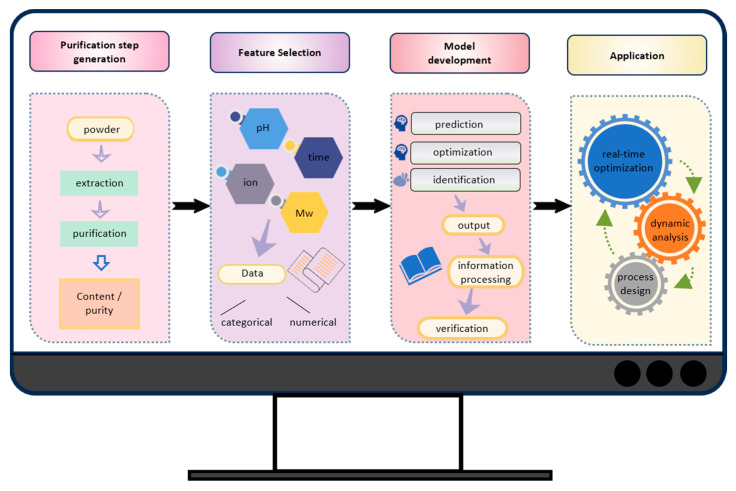
Integration of artificial intelligence modeling and purification technologies for the advanced purification of UPPs.

The integration of machine learning and artificial intelligence into polysaccharide purification represents a transformative approach to enhancing its efficiency, selectivity, and scalability (Figure 4) [74,75]. Traditional purification methods often depend on empirical optimization, which is both time-intensive and prone to variability. By leveraging machine learning techniques—such as supervised learning, reinforcement learning, and deep learning—the process can be streamlined through data-driven predictions, significantly reducing the amount of trial-and-error experimentation [76]. One of the most promising applications involves the use of artificial neural networks to optimize purification techniques such as chromatography, membrane filtration, and precipitation [77,78]. Artificial neural networks can analyze input variables—including the pH, temperature, solvent concentration, and flow rate—to predict the optimal purification conditions, reducing the need for manual adjustments and improving the reproducibility. When integrated with real-time sensor data, artificial neural networks enable dynamic process control, allowing for continuous feedback-based adjustments to maintain consistent product quality and yields [79].

Beyond process optimization, artificial neural networks can model intricate relationships between polysaccharide components, facilitating the selective isolation of specific polysaccharides—such as laminarin, fucoidan, and alginate—based on characteristics like their molecular weights, sulfation levels, and glycosidic linkages. Additionally, predictive modeling can anticipate equipment failures or inefficiencies, enabling proactive maintenance and reducing operational costs. Despite these advantages, challenges persist, including the need for extensive, high-quality datasets to train models effectively and the difficulty in interpreting artificial-intelligence-generated predictions. Addressing these issues will be crucial in fully harnessing artificial-intelligence-driven purification strategies, ultimately unlocking the full potential of polysaccharides for biomedical and industrial applications.

### 4.3. Economic and Environmental Considerations

From an economic standpoint, ethanol precipitation is efficient but demands high volumes of solvents, leading to increased processing expenses and environmental concerns. Moreover, the simultaneous precipitation of impurities lowers the product purity, requiring additional refinement [80]. Proper waste handling and recycling play a vital role in UPP purification. Byproducts, including leftover seaweed biomass and organic solvents, must be adequately managed to prevent environmental harm [81]. Possible recycling approaches involve recovering ethanol through distillation and repurposing residual seaweed as biofertilizer or animal feed, promoting sustainability [82].

Membrane filtration entails a considerable upfront investment in specialized membranes and filtration equipment, alongside continuous costs for maintenance, energy usage, and membrane replacement [83]. Efficiency is often hindered by fouling and clogging, which increase the operational expenses due to frequent cleaning and chemical treatments [84]. Furthermore, the loss of low-molecular-weight polysaccharides can impact the bioactive yield [85]. Nevertheless, membrane filtration remains a viable alternative to solvent-based purification by minimizing solvent use and simplifying processing [86].

Chromatographic purification poses significant economic and environmental challenges. Ion exchange chromatography requires expensive resins, buffers, and stringent operating conditions, driving up the production costs [87,88]. Similarly, SEC is labor-intensive and difficult to scale, making large-scale application costly. High solvent usage and prolonged processing times further limit their industrial feasibility [89]. From an environmental perspective, chromatographic techniques generate substantial liquid waste, including salt solutions and solvent residues, necessitating proper disposal. Implementing recycling measures such as regenerating ion exchange resins and optimizing solvent recovery can help to reduce waste and enhance sustainability [90].

In 2018, the global algae market was valued at approximately USD 717.14 million and is expected to grow to USD 1.37 billion by 2027 [91]. This industry covers a wide range of applications, including food and beverages, nutraceuticals, dietary supplements, personal care, animal feed, pharmaceuticals, chemicals, and biofuels. Notably, brands such as Innisfree and The Face Shop utilize seaweed polysaccharides in their cosmetic formulations. Likewise, Haerim Fucoidan Co., Ltd., headquartered in Wando-gun, Korea, produces seaweed extract capsules [92], while the Maruha Nichiro Corporation develops functional foods and supplements derived from wakame. The global seaweed industry is forecasted to reach USD 24.9 billion by 2028, with a projected annual growth rate of 7.51% from 2021 to 2028 [93].

### 4.4. Quality Control of UPPs

Maintaining the consistency, safety, and therapeutic effectiveness of UPPs demands stringent quality control measures. Physicochemical analysis plays a vital role, with the total carbohydrate content commonly assessed via the phenol–sulfuric acid method, while the molecular weight distribution is evaluated through gel permeation chromatography [94]. The monosaccharide composition, analyzed using high-performance liquid chromatography or gas chromatography–mass spectrometry, helps to identify key sugar residues like fucose, glucose, mannuronic acid, and guluronic acid, which influence bioactivity [71,95]. Additionally, structural characterization techniques such as Fourier transform infrared and nuclear magnetic resonance spectroscopy offer valuable insights into glycosidic linkages and sulfation patterns, both critical for biological functions [96,97].

Saccharide mapping is an advanced analytical approach that ensures UPPs’ consistency, authenticity, and bioactivity. This technique involves the enzymatic or mild chemical hydrolysis of polysaccharides into oligosaccharide fragments, followed by analysis to determine the structural composition, linkage arrangements, and sulfation profiles. Methods such as high-performance liquid chromatography, liquid chromatography–mass spectrometry, and capillary electrophoresis enable the precise identification of characteristic sugar motifs, ensuring uniformity across production batches. Furthermore, saccharide mapping helps to identify adulteration or structural alterations that may impact the biological activity. Since UPPs’ therapeutic potential is closely linked to their monosaccharide composition and glycosidic bonds, this method provides a reliable strategy for standardization.

## 5. Biological Activity

### 5.1. Antioxidant and Free Radical Scavenging Activity

Oxidative stress results from an imbalance between the generation of reactive oxygen species (ROS) and reactive nitrogen species and the body’s ability to counteract them with its antioxidant defenses [98]. These reactive molecules, produced during normal metabolic processes or triggered by external factors, are highly reactive and can damage essential cellular components such as lipids, proteins, and DNA. When oxidative damage is not controlled, it disrupts cellular functions and contributes to the development of various diseases, including cardiovascular disorders, diabetes, neurodegenerative conditions, liver and kidney diseases, and cancer [99,100]. Furthermore, oxidative stress plays a significant role in aging and age-related conditions, such as Alzheimer’s and Parkinson’s diseases, by accelerating neuronal degeneration [101]. The widespread impact of oxidative stress highlights the importance of effective antioxidant strategies to counteract its effects and prevent the progression of related diseases [102]. UPPs demonstrate strong antioxidant and free-radical scavenging activity, making them valuable in managing oxidative stress. These antioxidant properties are attributed to their distinctive structural features, including their sulfate groups, molecular weights, and specific glycosidic linkages, which enhance their capacity to neutralize ROS and minimize oxidative damage. Their mechanisms of action are diverse, encompassing free-radical scavenging, metal ion chelation, the inhibition of lipid peroxidation, and the modulation of endogenous antioxidant enzyme systems [103,104].

UPPs demonstrate direct antioxidant activity mainly by scavenging free radicals, including unstable molecules such as hydroxyl radicals (•OH) and superoxide anions (O_2_•^−^). This action helps to prevent oxidative damage to vital biomolecules, such as lipids, proteins, and DNA [105]. The efficiency of the radical scavenging activity is closely linked to the structural characteristics of UPPs, particularly their molecular weight. The low-molecular-weight fraction of fucoidan (<10 kDa) exhibited the highest 1,1-diphenyl-2-picrylhydrazyl scavenging activity, with a value of 1822.15 µg/mL (Trolox equivalents), compared to the high-molecular-weight fraction (>300 kDa), which showed 559.29 µg/mL, and the crude fucoidan extract, which demonstrated 650.56 µg/mL (both in Trolox equivalents) [25]. Similarly, fucoidan with a molecular weight below 10 kDa demonstrated hydroxyl radical scavenging activity of 86.98%, comparable to that of butylated hydroxyanisole (84.98%) and significantly higher than that of fucoidan with a molecular weight above 300 kDa, which exhibited 74.32% [106]. The sulfate content of fucoidan was found to be another structure–function relationship that influenced its antioxidant activity. The fucoidan with the highest sulfate content (25.19%) exhibited significantly higher antioxidant activity compared to other fucoidans (6.96% sulfate content) [107]. The sulfate groups may also stabilize the polysaccharide’s structure, allowing it to interact more effectively with reactive oxygen species.

Metal ions, like Fe^2+^, play a catalytic role in the Fenton reaction, resulting in the generation of highly reactive hydroxyl radicals (•OH) that can induce significant oxidative damage to lipids, proteins, and DNA [108]. Polysaccharides can suppress the Fenton reaction by chelating these metal ions, thereby minimizing hydroxyl radical production and interrupting oxidative chain reactions [109]. This chelation capability is linked to the structural characteristics of polysaccharides, such as hydroxyl, carboxyl, and sulfate functional groups, which interact with metal ions and stabilize them in less reactive states [110,111]. For instance, fucoidan from *Undaria pinnatifida* at a concentration of 500 μg/mL demonstrated iron-chelating activity that reached 73.55% [112]. Additionally, Silva et al. reported that fucoidan exhibited copper-ion-chelating activity reaching 25% [113].

UPPs also exhibit antioxidant effects indirectly by stimulating the body’s endogenous antioxidant defense systems. These systems include essential enzymes such as superoxide dismutase (SOD), catalase (CAT), and glutathione peroxidase (GSH-Px), which are crucial in neutralizing ROS and maintaining the redox balance [114]. Research has shown that polysaccharides can enhance the expression and activity of these enzymes, thereby improving the body’s resilience against oxidative stress [115]. For example, SOD facilitates the dismutation of superoxide anions (O_2_•^−^) into hydrogen peroxide, which is further broken down into water and oxygen by CAT or GSH-Px, preventing the accumulation of harmful ROS [116]. Furthermore, polysaccharides may promote the synthesis of glutathione, a critical non-enzymatic antioxidant that works alongside GSH-Px to detoxify peroxides [117]. A high-fat diet is strongly linked to oxidative stress and the dysregulation of endogenous antioxidant systems, contributing to the progression of various metabolic disorders [118]. Excessive dietary fat intake triggers the overproduction of ROS due to increased mitochondrial activity and lipid peroxidation, which overwhelms the body’s antioxidant defenses, including SOD, CAT, and GSH-Px [119]. The imbalance in ROS production and the body’s antioxidant defense mechanisms leads to oxidative stress, which plays a pivotal role in the development of obesity, insulin resistance, type 2 diabetes, and cardiovascular diseases [120]. Notably, sulfated polysaccharides derived from UPPs have demonstrated protective effects against HFD-induced metabolic syndrome in BALB/c mice by enhancing endogenous antioxidant systems [121]. Supplementation with varying doses of UPPs (low, medium, and high) resulted in a dose-dependent increase in SOD and CAT activity in HFD-fed mice. This indicates that UPPs effectively restored the liver’s antioxidant capacity, mitigating the oxidative stress caused by the HFD [121]. In an in vitro model, Chen et al. observed that UPPs enhanced the SOD levels and reduced intracellular ROS production in human renal proximal tubular epithelial cells subjected to oxalate-induced damage [122]. Similarly, Zheng et al. reported that UPPs increased the levels of GSH and SOD and the total antioxidant capacity in the hydrogen-peroxide-treated rat small intestinal epithelial cell line IEC-6 [123].

Although UPPs have garnered considerable attention as natural antioxidants, several challenges persist in unraveling their intricate mechanisms of action. The antioxidant properties of UPPs are strongly influenced by their molecular weights and sulfate groups, yet the exact molecular pathways through which they exert their effects, particularly in in vivo systems, remain incompletely understood [106,122]. Overcoming these hurdles requires the application of advanced analytical tools, such as spectroscopy, high-resolution imaging, and computational modeling, in conjunction with comprehensive biological assays [124,125]. Additionally, combining UPPs with other antioxidants, such as phenolic compounds and peptides, represents a promising approach to achieving synergistic effects against oxidative stress [5,126]. For instance, phenol–polysaccharide complexes merge the potent radical-scavenging activity of phenolic compounds with the structural stability and functional diversity of polysaccharides, enhancing the overall antioxidant performance [127]. Phenolic groups actively neutralize ROS, while the polysaccharide backbone improves phenols’ solubility, bioavailability, and controlled release [128]. These hybrid systems employ multifaceted antioxidant mechanisms, making them highly effective in mitigating oxidative damage within biological systems.

The antioxidant properties of UPPs make them promising candidates for a wide range of applications. In the nutraceutical field, they can be utilized in functional foods or dietary supplements to help combat oxidative stress. Moreover, their therapeutic potential includes the management of oxidative-stress-related diseases such as cardiovascular disorders, diabetes, and neurodegenerative conditions [129]. In the cosmetics industry, their ability to scavenge free radicals makes them an excellent ingredient for skincare products designed to reduce oxidative damage and prevent premature aging [130].

### 5.2. Immunomodulatory Activity

In recent years, the immunomodulatory properties of polysaccharides have attracted substantial interest due to their natural origins, diverse bioactivity, and broad therapeutic potential. Polysaccharides have demonstrated the ability to modulate both innate and adaptive immune responses, making them promising candidates in addressing immune-related conditions such as infections, inflammation, autoimmune diseases, and cancer [131]. Their dual functionality—enhancing immune activities like macrophage activation, cytokine production, and antibody synthesis, while simultaneously dampening excessive inflammatory responses—has established polysaccharides as significant agents in immunology and biotechnology [132].

Polysaccharides influence the innate immune system by activating key immune cells, including macrophages, dendritic cells (DCs), and natural killer (NK) cells [94,133]. Macrophages, as part of the body’s first line of defense, are stimulated by polysaccharides to release cytokines such as interleukin-6 (IL-6), tumor necrosis factor-alpha (TNF-α), and nitric oxide (NO), which play critical roles in pathogen elimination and inflammation control [134,135]. For instance, UPPs, characterized by a predominant xylose composition and uronic acid content of 13.08%, significantly enhanced the proliferation and pinocytic activity of RAW264.7 macrophage cells. Additionally, they upregulated the mRNA expression of key immune mediators, including inducible nitric oxide synthase (iNOS), TNF-α, IL-6, and IL-1β [136]. IL-6 is a versatile cytokine that regulates acute-phase reactions, supports B-cell differentiation, and modulates inflammation, while IL-1β and TNF-α play crucial roles in recruiting immune cells to infection or injury sites and amplifying immune responses. Furthermore, research by Liu et al. revealed that UPPs markedly promotes dendritic cell maturation both in vitro and in vivo. In vitro studies demonstrated that low-molecular-weight fractions of UPPs increased the expression of dendritic cell maturation markers such as CD40, CD86, MHC I, and MHC II, further supporting their immunomodulatory potential [137].

Polysaccharides are recognized for their ability to modulate Toll-like receptor (TLR) signaling pathways, which play a pivotal role in the innate immune system by detecting and responding to pathogen-associated molecular patterns [138]. By interacting with specific TLRs, such as TLR2, TLR4, TLR8, and TLR9, polysaccharides can either activate or regulate immune responses, influencing both pro-inflammatory and anti-inflammatory pathways [139]. For instance, the pretreatment of dendritic cells with TAK-242, a TLR4 inhibitor, significantly decreased the expression of maturation markers (CD40 and CD86) and the secretion of cytokines (IL-12 and TNF-α) induced by low-molecular-weight fucoidan, indicating that its immune-stimulating effects are mediated via TLR4 [137]. When UPPs bind to TLR4 on macrophages or dendritic cells, they trigger the MyD88-dependent signaling cascade, leading to the activation of key signaling molecules such as IκB kinase (IKK) and various members of the MAP kinase (MAPK) family, including ERK, JNK, and p38 [140]. This cascade culminates in the activation of NF-κB, which translocates its subunits into the nucleus, where they promote the transcription of pro-inflammatory cytokines and other immune mediators [141]. Concurrently, MAPK signaling enhances these effects by regulating cytokine production and promoting cell survival [142]. These signaling pathways are critical in orchestrating the immune response to infections and injuries, further underscoring the immunomodulatory potential of polysaccharides.

Future research on UPP-based immunomodulation will aim to deepen our understanding of the structure–function relationship, particularly how specific functional groups—such as sulfate groups and uronic acid residues—interact with immune receptors and influence downstream signaling pathways. While considerable progress has been made in identifying the bioactivity of polysaccharides, the precise mechanisms through which they interact with immune receptors remain incompletely understood. Moreover, modifying the chemical structures of UPPs, such as altering their molecular weights or adding specific functional groups, presents an opportunity to fine-tune their bioactivity, solubility, and receptor-binding affinity. These structural adjustments could enhance the therapeutic potential of polysaccharides while minimizing possible side effects. Consequently, UPPs could be more effectively applied across a broader range of clinical settings, including immunotherapy, cancer treatment, and the management of inflammatory diseases.

### 5.3. Anticancer Activity

Polysaccharides have attracted significant interest as bioactive functional compounds in cancer prevention and treatment due to their wide-ranging biological properties and minimal or non-toxic nature. In contrast to traditional chemotherapy drugs, which often lead to severe adverse effects, polysaccharides provide a more targeted mechanism by enhancing immune responses, suppressing tumor cell growth, and triggering apoptosis in cancer cells [143]. UPPs have the ability to selectively induce cell death in malignant cells while causing little to no harm to normal, healthy cells. Their cytotoxic effects are primarily linked to their capacity to interfere with essential cellular functions such as proliferation, metabolism, and survival pathways. For example, UPPs exhibited direct anticancer potential against the MCF7 human breast cancer cell line by reducing cell proliferation and migration in a dose-dependent manner at concentrations between 25 and 200 μg/mL [144]. Moreover, UPPs have been found to induce apoptosis in ovarian cancer cells and inhibit cell proliferation, migration, and invasion by modulating the Hedgehog signaling pathway [145]. Lee et al. reported that UPPs induced cell cycle arrest at the G0/G1 phase and triggered apoptosis in human PC-3 prostate cancer cells by suppressing the Akt/GSK-3β/β-catenin signaling pathway. Additionally, their research revealed that UPPs (200–400 mg/kg) significantly reduced the tumor size and volume in a xenografted mouse model [146].

Furthermore, UPPs can modulate and enhance various components of the immune system, strengthening the body’s natural defense against tumors. They have been found to activate essential immune cells, including macrophages, dendritic cells, and natural killer (NK) cells, which are vital in identifying and destroying cancer cells. By stimulating the release of pro-inflammatory cytokines such as TNF-α, IL-1, and IL-6, UPPs help to establish an immune-responsive environment that inhibits tumor progression. Their antitumor effects have been observed in sarcoma 180 subcutaneous xenograft-bearing mice, where they activated immune cells and boosted the production of pro-inflammatory cytokines [147].

Future advancements in utilizing UPPs for cancer therapy are expected to emphasize the integration of nanotechnology and cutting-edge drug delivery systems to maximize their therapeutic potential. Although UPPs demonstrate strong antitumor properties through direct cytotoxic effects and immune regulation, their clinical application is often hindered by challenges such as limited bioavailability and inefficient tumor targeting. Nanotechnology presents innovative strategies to address these limitations by encapsulating polysaccharides into nanoparticles, liposomes, or hydrogels, thereby enhancing their stability, controlled release, and tumor selectivity. Functionalized nanoparticles can be engineered to improve cellular uptake and preferentially accumulate in tumor tissues via passive targeting mechanisms, such as the enhanced permeability and retention effect, or through active targeting approaches involving surface modifications with ligands that specifically bind to cancer-related receptors. These advancements in drug delivery technology could significantly enhance the therapeutic effectiveness of UPPs, increasing their potential for clinical applications in cancer treatment.

### 5.4. Prebiotic Activity and Mucosal Barrier

The gut microbiota plays an essential role in maintaining host health by influencing various physiological processes, such as digestion, metabolism, immunity, and the integrity of the intestinal barrier [148]. Disruptions to the gut microbiome can lead to a range of health issues, including inflammatory bowel disease, metabolic disorders, and neurodegenerative diseases [149]. In recent years, prebiotics—non-digestible food components that selectively promote the growth or activity of beneficial microorganisms—have gained attention as a strategy to support a balanced microbiome and improve intestinal health [150,151,152].

UPPs have been shown to modulate the growth and activity of beneficial gut microbes. They serve as substrates for the gut microbiota, fostering the proliferation of health-promoting bacteria while inhibiting the growth of pathogenic species. In high-fat-diet-induced metabolic dysfunction, supplementation with UPPs was associated with a significant increase in specific beneficial bacteria, such as *Bacteroides acidifaciens* and *Bacteroides ovatus* [153]. Additionally, in an in vitro simulation of distal colonic fermentation, UPPs promoted the growth of the Bacteroidetes phylum [154]. Species of *Bacteroides* are known for their ability to degrade a broad range of polysaccharides, aided by their extensive repertoire of carbohydrate-active enzymes [155]. These enzymes catalyze the hydrolysis of glycosidic bonds in carbohydrates, allowing microbes to break down complex polysaccharides into simpler sugars that can then be fermented into beneficial metabolites [156]. Furthermore, Yang et al. reported that UPP treatment in high-fat-diet-fed mice increased the abundance of beneficial bacteria such as *Akkermansia muciniphila* and *Lactobacillus* [157]. *A. muciniphila* has been shown to provide numerous beneficial effects on host health, particularly in the context of metabolic and gastrointestinal diseases [158]. Several studies have highlighted its potential to enhance intestinal barrier function by boosting mucus production and promoting the expression of tight junction proteins, which are critical in maintaining gut integrity [159]. In addition to their direct effects on microbial populations, the modulation of beneficial gut microbes by UPPs can enhance the overall microbial ecosystem by promoting microbial diversity. A more diverse microbiota is linked to a healthier gut and improved metabolic outcomes, as it helps to bolster the resilience of the gut microbiome against disturbances, such as those induced by a poor diet, antibiotic use, or stress [160]. By fostering the growth of beneficial microbes and maintaining a balanced microbial community, polysaccharides can play a crucial role in supporting gut health, preventing inflammation, and strengthening the protective functions of the intestinal barrier.

UPPs are not digested in the upper gastrointestinal tract but are fermented in the large intestine by a diverse array of microbes. These microbes break down the complex carbohydrates in UPPs into short-chain fatty acids (SCFAs), including acetate, propionate, and butyrate [161]. SCFAs are of particular interest due to their wide-ranging beneficial effects on gut health. Acetate, the most abundant SCFA in the colon, serves as an energy source for epithelial cells and plays a role in regulating intestinal motility and fluid absorption [162]. Propionate is involved in maintaining gut barrier function and modulating inflammation [163], while butyrate, the primary energy source for colonocytes (intestinal epithelial cells), is crucial in preserving gut barrier integrity [164]. SCFAs have also been shown to influence appetite regulation, fat storage, and insulin sensitivity, thereby impacting the development and progression of obesity. Treatment with UPPs in high-fat-diet-fed mice significantly reduced body weight gain and the accumulation of epididymal and abdominal adipose tissue by increasing SCFA production [153]. Zhang et al. found that treatment with sulfate polysaccharides and alginate derived from *U. pinnatifida* significantly promoted the production of acetate and propionate in high-fat-diet-fed mice [165]. SCFAs regulate appetite by activating receptors such as GPR41 and GPR43, which are expressed in various tissues, including the gut, pancreas, and brain [166]. The activation of these receptors by SCFAs stimulates the release of satiety hormones like peptide YY and glucagon-like peptide-1, which help to reduce food intake and induce feelings of fullness [167]. Additionally, SCFAs improve insulin sensitivity and glucose homeostasis, processes that are often disrupted in individuals with obesity. This metabolic regulation plays a key role in reducing the risk of developing type 2 diabetes, a common comorbidity of obesity [168].

Mucus plays a critical role as a protective barrier in the gastrointestinal tract, primarily composed of mucins—high-molecular-weight glycoproteins that offer both mechanical and chemical defense [169]. Among the various types of mucins, Mucin2 (MUC2) is the most abundant and essential in maintaining the intestinal mucus layer, which acts as the first line of defense against pathogens, toxins, and physical injury [170]. MUC2 is predominantly secreted by goblet cells, which are scattered across the intestinal lining. These cells are responsible for synthesizing, packaging, and secreting mucins, forming a viscous layer that traps pathogens and particulate matter while facilitating their clearance from the gut [171]. The secretion of MUC2 is regulated by several signaling pathways, ensuring that mucus production remains sufficient to maintain the intestinal barrier without excessive buildup, which could hinder nutrient absorption [172]. Histological studies have demonstrated that UPP treatment in fiber-deficient mice led to an increase in the density of glycoproteins and goblet cells, as well as elevated levels of MUC2 and GAL3ST2 expression [173,174]. Additionally, UPP treatment significantly boosted the number of goblet cells in the middle and distal intestines compared to the control group, while reducing the abundance of mucin-degrading bacteria like *Mucinivorans* [175].

Tight junctions are specialized protein complexes that play a vital role in maintaining the integrity of the intestinal epithelial barrier [176]. They regulate the selective permeability of the gut epithelium, controlling the passage of ions, water, and small molecules, while preventing the leakage of harmful pathogens and toxins from the gut lumen into the bloodstream [177]. Tight junctions are primarily composed of transmembrane proteins such as occludin, claudins, and junctional adhesion molecules, which interact with intracellular scaffolding proteins like zonula occludens (ZO)-1, ZO-2, and actin filaments [178]. Together, these proteins form a dynamic barrier that ensures gut health and prevents intestinal permeability (leaky gut). The disruption of tight junctions, often caused by factors such as a high-fat diet or chronic inflammation, can lead to increased intestinal permeability and contribute to various health issues, including metabolic disorders, inflammatory bowel disease, and other systemic conditions [179]. A high-fat diet, particularly one that is rich in saturated fats and low in fiber, can promote systemic inflammation, impair tight junction protein expression, and compromise intestinal barrier function [180]. In contrast, polysaccharides like those derived from UPPs have been shown to support the integrity of the intestinal barrier. In studies with fiber-deficient mice, high doses of UPPs (400 mg/kg body mass/day) significantly increased the expression levels of key tight junction proteins, including occludin, ZO-1, and claudin-3. These findings suggest that UPPs can help to restore tight junction integrity, potentially mitigating the negative effects of dietary-induced inflammation and enhancing gut barrier function. This mechanism further underscores the beneficial role of UPPs in maintaining intestinal health and preventing intestinal permeability [123].

While it is known that UPPs can support the growth of beneficial gut bacteria, the exact strains that effectively ferment UPPs remain largely unidentified. This gap in knowledge makes it difficult to pinpoint which microbial species are most capable of metabolizing these polysaccharides to generate bioactive metabolites that can enhance gut barrier function, promote mucus production, or modulate the immune response. Moreover, the specific metabolites produced by these bacterial strains, and how they interact with the gut epithelium to influence the integrity of the mucus layer, are not yet fully elucidated. Understanding the metabolic pathways involved in the fermentation of UPPs will be crucial in identifying the key metabolites responsible for modulating the mucus barrier and enhancing intestinal integrity.

### 5.5. Anti-Hyperglycemic Effects

Diabetes mellitus is a long-term metabolic condition marked by sustained high blood glucose levels, resulting from insulin resistance, insufficient insulin production, or a combination of both [181]. Conventional treatments, including insulin therapy and oral hypoglycemic agents, often come with side effects such as weight gain, gastrointestinal disturbances, and the risk of hypoglycemia [182]. As a result, there is growing interest in natural alternatives, particularly polysaccharides derived from plants, fungi, and marine organisms, due to their diverse biological properties and minimal adverse effects [183]. UPPs have been shown to exert anti-hyperglycemic effects through multiple mechanisms, including the inhibition of carbohydrate-digesting enzymes, modulation of insulin signaling pathways, regulation of glucose transporters, and protection of pancreatic β-cells from oxidative stress and inflammation [96,184].

UPPs demonstrate anti-hyperglycemic properties primarily through the modulation of glucose metabolism. UPPs regulate postprandial blood glucose levels by inhibiting key enzymes involved in carbohydrate digestion. Notably, UPPs have been shown to inhibit α-amylase, amyloglucosidase, and α-glucosidase, with IC_50_ values of 0.190, 0.280, and 0.137 mg/mL, respectively. Among these enzymes, α-amylase and amyloglucosidase act as uncompetitive inhibitors, while α-glucosidase is a competitive inhibitor [22]. α-Amylase breaks down complex carbohydrates into smaller oligosaccharides, which are further hydrolyzed by α-glucosidase into glucose, allowing its absorption in the small intestine [185,186]. By competitively inhibiting these enzymes, UPPs delay the release and absorption of glucose, preventing sharp increases in blood sugar levels following meals. In addition to reducing glucose absorption, UPPs also promote glucose uptake and utilization in peripheral tissues, such as skeletal muscle and adipose tissue. Previous research suggests that UPPs activate key signaling pathways, including the Akt and AMP-activated protein kinase pathways [187], which are crucial for glucose metabolism. The activation of these pathways leads to the increased expression and translocation of glucose transporter 4, a critical protein involved in facilitating glucose entry into cells. By enhancing glucose transporter 4-mediated glucose uptake, UPPs improve insulin sensitivity and boost cellular glucose utilization, thereby lowering circulating blood glucose levels [188].

UPPs also play a key role in improving insulin sensitivity, which is vital in regulating blood glucose levels, particularly in individuals with insulin resistance. A prior in vitro study demonstrated that UPPs enhanced glucose uptake in normal 3T3-L1 adipocytes and reinstated insulin-stimulated glucose uptake in adipocytes with obesity-induced insulin resistance. This insulin resistance was induced by exposing hypertrophied 3T3-L1 cells to conditioned media from RAW 264.7 macrophages [189]. Furthermore, in vivo studies underscored UPPs’ potential to mitigate insulin resistance, enhance glucose tolerance, and promote the secretion of glucagon-like peptide-1 (GLP-1) in a type 2 diabetes rat model [184]. GLP-1 is an incretin hormone released from the intestines following food intake. It promotes insulin secretion in a glucose-dependent manner, meaning that it stimulates insulin release when blood glucose levels are elevated, but not when they are normal or low [190,191]. Moreover, GLP-1 inhibits the secretion of glucagon from the pancreas, a hormone that typically raises blood glucose by stimulating hepatic glucose production [192].

Pancreatic islet cells, especially insulin-secreting β-cells, are essential in maintaining blood glucose homeostasis [193]. In diabetes, persistent hyperglycemia and inflammation contribute to oxidative stress, leading to β-cell dysfunction and impaired insulin secretion. This deterioration is a major factor in the progression of both type 1 and type 2 diabetes [194]. Research has demonstrated that UPPs could mitigate pancreatic islet cell damage and enhance β-cell function in hyperglycemic mice induced by a high-fat diet and streptozotocin, highlighting their potential protective role in diabetes management [53].

## 6. Conclusions

UPPs have garnered significant interest due to their wide-ranging biological activity, including antioxidant, immunomodulatory, anticancer, and gut health-enhancing properties. Recent progress in extraction and purification techniques—such as EAE, UAE, and MAE—has improved their yields, while advanced chromatographic methods have enhanced their purity and fractionation. Given their potent bioactivity, UPPs present strong potential for use in functional foods, nutraceuticals, and pharmaceuticals. As natural antioxidants and immunomodulatory agents, they can be incorporated into dietary supplements, functional beverages, and pharmaceutical formulations aimed at combating oxidative-stress-related disorders. Their prebiotic properties and ability to regulate the gut microbiota further suggest potential applications in digestive health products, while their anticancer effects create opportunities for therapeutic interventions.

Despite these promising prospects, further research is needed to enable large-scale production. Future efforts should prioritize the development of continuous-flow MAE and UAE systems to improve their efficiency while preserving polysaccharides’ bioactivity. Pilot-scale studies and techno-economic evaluations will be essential in assessing the feasibility of industrial-scale production while minimizing environmental impacts. Additionally, integrating artificial intelligence into the purification process could transform the efficiency and reproducibility. Artificial-intelligence-driven predictive models can optimize the purification parameters, such as the ion exchange chromatography conditions, ultrafiltration membrane selection, and gradient elution strategies, reducing the reliance on trial-and-error methods. Machine learning algorithms and artificial neural networks could also refine real-time spectral analysis, allowing for the automated identification and fractionation of high-purity, bioactive polysaccharides. Ensuring the safety of UPPs is a significant challenge in commercial applications. Major concerns include variations in bioactivity caused by source heterogeneity, residual solvents or contaminants from extraction processes, and potential risks of allergenicity or immunogenic reactions. To confirm their safety, thorough toxicological evaluations, such as acute and chronic toxicity assessments, allergenicity testing, and pharmacokinetic studies, are crucial in defining their safety profiles. Further research should also aim to clarify the molecular mechanisms underlying the biological effects of UPPs. While their antioxidant, anti-inflammatory, immunomodulatory, and anticancer properties are well documented, their precise roles in cellular signaling pathways remain insufficiently understood. In-depth mechanistic studies using both in vitro and in vivo models will be crucial to unlocking their full therapeutic potential. With continued advancements, UPPs hold significant promise as valuable bioactive compounds for health and medical applications.

## Data Availability

No new data were created or analyzed in this study. Data sharing is not applicable to this article.
